# The Integration of Surgical Quality Assurance Within the Life Cycle of a Clinical Trial

**DOI:** 10.1097/SLA.0000000000007038

**Published:** 2026-02-25

**Authors:** Sheraz R. Markar, Nainika Menon, Maria Bencivenga, Suzanne Gisbertz, George B. Hanna

**Affiliations:** 1 Nuffield Department of Surgical Sciences, University of Oxford, Oxford, United Kingdom; 2 Division of Esophageal and Gastric Cancer Surgery, Department of Surgery, Dentistry, Paediatrics and Gynaecology, University of Verona, Italy; 3 Department of Surgery, Amsterdam UMC location University of Amsterdam, Amsterdam, The Netherlands; 4 Cancer Treatment and Quality of Life, Cancer Center Amsterdam, Amsterdam, The Netherlands; 5 Department of Surgery and Cancer, Imperial College London, London, United Kingdom

**Keywords:** clinical trials, surgical quality assurance

The emergence of surgical quality assurance (SQA) as a core principle in the conduct of randomized controlled trials (RCTs) provides a unique opportunity to:

- Standardize and assure the quality of the surgical intervention and participating surgeons.
- Recalibrate the sample size to ensure the RCT reflects “real-world clinical practice.”
- Develop and validate competency assessment tools (CATs) that can be used to facilitate the implementation of novel surgical procedures upon completion of the RCT.

## CHALLENGE WITH SURGICAL RCTs

RCTs still represent the highest level of available evidence when evaluating surgical procedures. A major obstacle in undertaking robust RCTs is that, unlike a pharmaceutical trial that provides patients with a medication, surgical performance is highly variable both within and outside of RCTs. This leads to 2 major challenges: firstly, if the quality of surgery has a large degree of heterogeneity within the RCT, then this will dilute the expected effect size of the intervention and ensure the RCT is underpowered, and an unreliable result ensues. Secondly, more commonly, surgical RCTs are conducted in centers of excellence, and thus, when these complex surgical techniques are implemented more broadly nationally or internationally, the heterogeneity in surgical performance often ensures the trial result is not replicable in “real-world” clinical practice, and thus, the RCT lacks external validity. A good example of this is minimally invasive esophagectomy (MIE), with the Dutch TIME RCT conducted in 5 international centers showing a substantial reduction in pulmonary complications associated with a minimally invasive approach over open surgery.^1^ However, immediately following the RCT when MIE was implemented nationally across the Netherlands, MIE was associated with an increase in pulmonary complications and reoperations, and thus the TIME RCT has no external validity in real-world clinical practice.^2^

## PRINCIPLES AND VALUES OF SQA

There are 3 main components to SQA when conducted within RCTs: (1) standardization of surgical technique (SST), (2) credentialing of surgical experience (CSE), and (3) monitoring of surgical performance (MP). The principal aims of SQA programs are to reduce variability in surgical performance as a potential confounder within RCTs and to mitigate potential learning curve effects associated with the adoption of new surgical procedures within RCTs. We have previously shown that robust SQA within RCTs can reduce variability and improve patient outcomes in oesophagectomy, gastrectomy, and bariatric surgery.^3,4^

As we move toward an era of increased minimally invasive techniques, including robotic-assisted surgery across the breadth of surgical procedures, this provides a unique opportunity to collect intraoperative videos and assessment of surgical performance.^5^ There has been an exponential growth in the development of CATs based on intraoperative video analysis, which are utilized to grade the quality of surgical performance. Often, these CATs are created through an expert Delphi consensus process, and occasionally, inter- and intraobserver reliability is assessed using a small sample size of intraoperative videos. Thus, there often remains a healthy amount of surgical skepticism regarding the true validity of these tools when applied inside and outside a clinical trial.

Furthermore, analysis of intraoperative videos is labor-intensive and cumbersome, which has been an Achilles heel of CAT clinical integration. However, machine learning techniques and the use of artificial intelligence (AI) will have a real role in SQA in the future. Initially, this will be through surgical phase recognition, allowing raters to identify and score critical steps of the procedure in an abbreviated fashion. Following this, the natural next step will be to use AI algorithms to rate and train surgical performance using the developed CATs, which has been done for less complex procedures, including laparoscopic cholecystectomy.^6^ Further, the ultimate goal will be to integrate AI into SQA processes to facilitate intraoperative guidance and postoperative feedback to optimize surgical performance both within and outside of RCTs. It is also important to consider the impact of robust SQA processes across real-world clinical practice, with the downsides being exclusion of lower-volume or resourced surgeons and settings, specifically from low-income countries without access to video recording equipment. Further, if SQA is too tightly controlled within an RCT, it may be unreproducible within real-world clinical practice, unless a clear line of sight is developed from the RCT to clinical practice.

## INTEGRATION OF SQA WITHIN THE LIFECYCLE OF AN RCT

**FIGURE 1:**
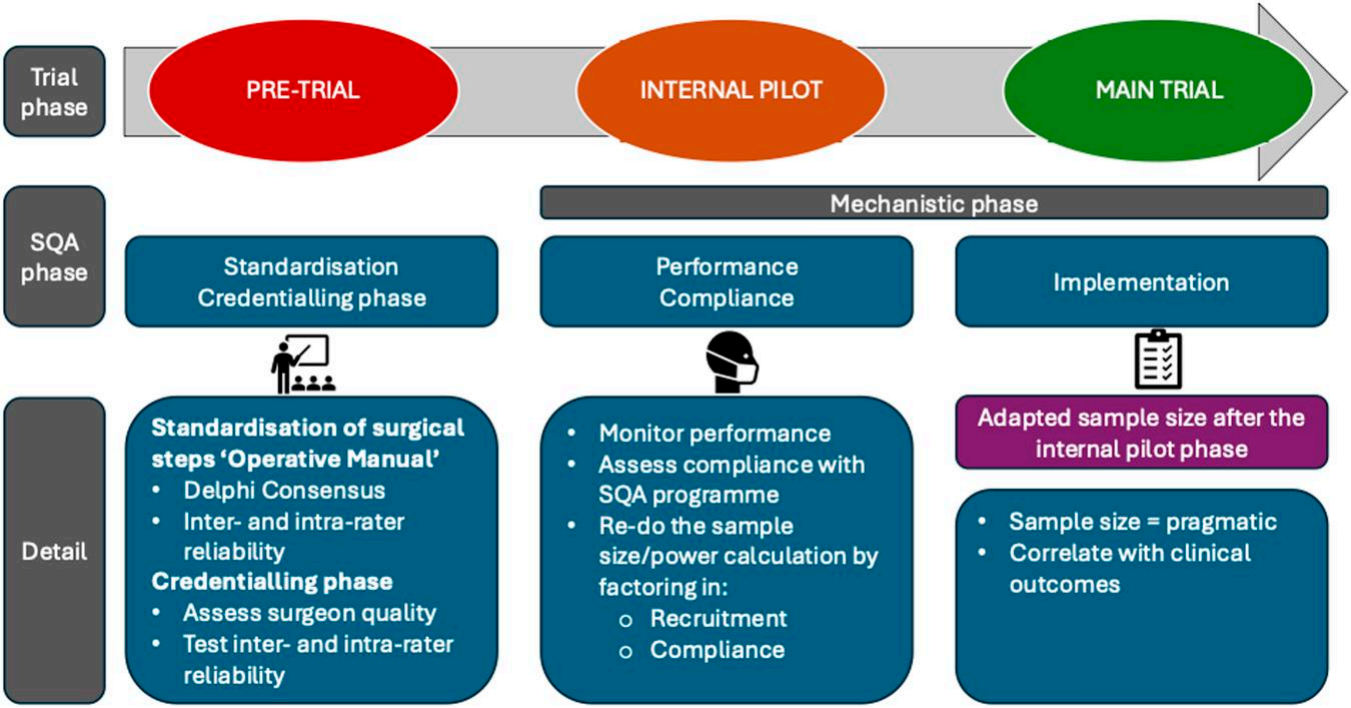
Infographic demonstrating the integration of surgical quality assurance within the lifecycle of a randomised controlled trial.

RCTs provide a unique opportunity to develop and validate CATs to improve the quality of surgery both inside and outside a trial setting. Within the pretrial stage of an RCT, the SST phase of an SQA program often includes an expert Delphi consensus with mandatory and optional steps defined, along with potential technical errors identified during development of the proposed CAT for the trial. Following the development of the CAT, a small series of intraoperative videos is analyzed by independent assessors to evaluate inter- and intrarater reliability. Within the pretrial stage of an RCT, the CSE phase of the SQA program ensures all participating surgeons perform the procedure to a sufficient standard to enter the trial. When CSE is performed through analysis of intraoperative videos using a developed CAT, this provides a robust quantitative metric and acts as a gatekeeper for surgeons entering the RCT. Further, this provides an additional opportunity to test inter- and intrarater reliability of the developed CAT through independent rater assessment before the further stages of the RCT (Fig. 1).

Commonly, in large surgical RCTs, there is an internal pilot study, primarily utilized to assess recruitment rates and demonstrate to the funding body the feasibility of proceeding to the main trial. This phase of an RCT represents a unique and commonly unused opportunity to monitor performance using the developed CAT and assess compliance with the agreed surgical protocol for the trial. There will inevitably be some heterogeneity in surgical performance, and historically, this was ignored entirely within RCTs. More recent trials have tried to control this to such a level that the trial is no longer representative of the average surgeon performing the technique, and thus the trial lacks external validity. The next step in the evolution of SQA is to accept that the CSE phase has identified an acceptable group of surgeons performing the technique well; however, within the trial, there will still be a degree of variation in performance, which reflects real-world clinical practice. Collecting and analyzing intraoperative videos during the internal pilot phase of the RCT will quantify this variability in surgical performance among credentialled surgeons, and is representative of the likely compliance within the main trial. Thus, at this stage upon completion of the internal pilot, we advocate for a recalculation of the sample size with the expected compliance to the surgical protocol, as this is more likely to be representative of real-world clinical practice and the true expected effect size of the intervention (Fig. 1). Upon completion of the RCT, monitoring of performance with assessment of intraoperative surgical performance using the developed CAT can then be compared with clinical outcomes, and thus robustly evaluate content validity of the CAT. Then, following the RCT, a validated CAT has been developed, which can be used to train and safely disseminate the complex surgical procedure under evaluation within the RCT.

## CONCLUSION

Chief investigators conducting surgical RCTs must first accept that surgery is not a medication and that there is inherent variation in performance and the resulting effect size of the intervention. SQA must be integrated within the RCT lifecycle to ensure an acceptable level of performance within the trial and to reduce patient harm during any potential learning curve effects associated with the adoption of a new interventional technique. The development and robust validation of CATs within well-conducted surgical RCTs will serve as a valuable educational tool when it comes to the broader implementation of the surgical technique in nonparticipating centers outside the RCT upon completion.
